# Relationship between Total Lymphocyte count (TLC) and CD4 count among peoples living with HIV, Southern Ethiopia: a retrospective evaluation

**DOI:** 10.1186/1742-6405-5-26

**Published:** 2008-12-22

**Authors:** Deresse Daka, Eskindir Loha

**Affiliations:** 1 Faculty of Medicine, Hawassa University, Hawassa, Ethiopia; 2 Faculty of Public Health, Hawassa University, Hawassa, Ethiopia

## Abstract

**Background:**

CD4 count is a standard measure of immunodeficiency in adults infected with HIV to initiate and monitor highly active antiretroviral therapy; however, it may not be feasible in resource poor countries. There is a need to have another marker of immunodeficiency that is less resource demanding.

**Objective:**

The objective of this study was to assess the relationship between total lymphocyte count and CD4 count in one of the resource poor countries, Ethiopia.

**Methods:**

This was a retrospective evaluation. A total of 2019 cases with total lymphocyte and CD4 counts from three hospitals (Yirgalem, Hossana and Arba-Minch) were included in the study. Pearson correlation, linear regression and Receiver Operating Characteristic (ROC) were used.

**Result:**

For adults, the sensitivity, specificity, positive and negative predictive values of TLC < 1200 cells/mm^3 ^to predict CD4 count < 200 cells/mm^3 ^were 41%, 83.5%, 87.9% and 32.5%, respectively. For subjects aged less than 18 years, these values were 20.2%, 87%, 82% and 27.1%, respectively. A TLC ≤ 1780 cells/mm^3 ^was found to have maximal sensitivity (61%) and specificity (62%) for predicting a CD4 cell count of < 200 cells/mm^3^. Meanwhile, a TLC ≤ 1885 cells/mm^3 ^would identify only 59% of patients with CD4 count of < 350 cells/mm^3^(sensitivity, 59%; and specificity, 61%). The combined sensitivity and specificity for patients above 40 years of age was greater.

**Conclusion:**

Our data revealed low sensitivity and specificity of TLC as a surrogate measure for CD4 count.

## Background

It is estimated that 32.2 million people worldwide were living with HIV at the end of 2007. Meanwhile, 2.1 million lost their lives to AIDS, and 2.5 million became newly infected with HIV in the same year [1]. The proportion of people who have become infected with HIV is believed to have peaked in the late 1990s and stabilized subsequently; nonetheless the incidence is still increasing in several countries [2].

In Sub-Saharan Africa, the estimated number of adults and children living with the virus at the end of 2007 was 22.5 million, nearly 70% of the global share [1]. Meanwhile this is the region where there is resource limitation to address the problem, scarcity of CD4 counter to initiate highly active antiretroviral therapy (HAART), for instance. The determination of CD4 count has become a standard measure of immunodeficiency in adults infected with HIV in resource rich areas where the burden of the pandemic is low [3]. Cognizant of this problem, the current guidelines from World Health Organization (WHO) acknowledge that total lymphocyte count (TLC) may be used to make treatment decision in resource poor settings when CD4 count is not available and patients are mildly symptomatic [4].

The rationale for the WHO's recommendation is that most studies concluded a decline in TLC was strongly correlated with a decline in CD4 count, though there were some discrepancies [5-10]. On the other hand, there is a recent report warned that TLC < 1200 cells/mm^3 ^was not optimal for identifying patients requiring HAART since it showed low sensitivity and specificity to predict CD4 count below 200 cells/mm^3 ^[10,11]. This necessitates further study on the relationship between TLC and CD4. Therefore, the objective of this research was to assess the relationship between total lymphocyte count (TLC) and CD4 count in one of the resource poor countries, Ethiopia.

## Methods

A retrospective evaluation was carried out in three hospitals (Yirgalem, Arba-Minch and Hossana) in the southern part of Ethiopia. Collating data was burdensome as we reviewed 3120 antiretroviral treatment (ART) and pre-ART cards (Yirgalem); 2180 ART and pre ART cards (Arba-Minch); and more than 20 000 non-ART, ART and pre-ART cards (Hossana). The total number of cases with complete data on TLC and CD4 counts was 2019 of which 750, 650 and 619 were from Yirgalem, Arba-Minch and Hossana hospitals, respectively. The year of the data extends from 2003 to 2008. All cases were hospital patients. In all hospitals, TLC and CD4 counts were determined using Cell Dyne automated machine from ABBOTT, USA.

SPSS 15 was used to analyze the data. Linear regression was carried out. As the CD4 and TLC values were log transformed to maintain normality, 100(e^*β*ln(1.01) ^- 1)[12] was used to interpret the regression coefficient *β*, and expressed as percentage points. Pearson correlation coefficient was also reported.

Receiver Operating Characteristic (ROC) was used to determine the cut-off points with best sensitivity and specificity combination. Area under the ROC curve (AUC) was also used to compare the combined sensitivity and specificity among different categories of the study subjects.

Ethical clearance was obtained from College of Health Sciences, Hawassa University-Institutional Ethical Review board, and permission was sought from each hospital.

## Results

A total of 2019 subjects were included in this study, among which 1064 (53%) were females. The mean (standard deviation) age was 32.4 (9.4) years (ranging from 5–65 years), and the majority, 1707 (85%) were below the age of 40 years. Three fourth of the study subjects had CD4 count less than 200 cells/mm^3^, and 97% had a count of less than 350 cells/mm^3^. The mean (standard deviation) of CD4 and TLC counts were 145.1 (94.9) cells/mm3 and 1734.1 (880.9) cells/mm3 for subjects aged 18 years and above, and for those under the age of 18 years, the figures were 200.4 (170.6) cells/mm3 and 3700 (942.9) cells/mm3, respectively.

**Figure 1:**
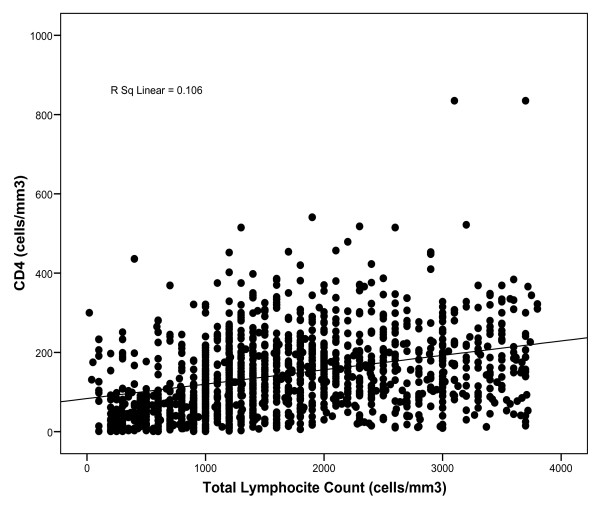
**Relationship between CD4 and TLC counts**.

The correlation coefficient *r *for lnCD4 and lnTLC was .398 (p < .001). The linear regression coefficient (*β*) was 0.61; that is for each 1% increase in TLC there was 0.61% increase in CD4 count. However, the model was capable of explaining only 16% (coefficient of determination-R^2 ^adjusted) of the variation. Figure 1 shows the relationship between CD4 and TLC counts using the original scales of measurement (R^2 ^= 0.1, r = 0.33, p < 0.001).

Mean CD4, sensitivity, specificity, positive predictive value (PPV) and negative predictive value (NPV) for different levels of TLC cut-off values among those who were less than 18 years of age and adults are depicted in table 1.

**Table 1:** Different cut-off values of TLC predicting CD4 < 200 cells/mm3 for subjects aged 18 years and above, and less than 18 years.

**TLC cut-off values (cells/mm3)**	**Mean CD4 (cells/mm3)**		**Sensitivity**		**Specificity**		**PPV**		**NPV**	
	
	< 18	≥ 18	< 18	≥ 18	< 18	≥ 18	< 18	≥ 18	< 18	≥ 18
1000	154.5	86.6	14.9	21.8	87.0	95.3	77.0	93.2	25.8	29.3
1200	150.1	99.2	20.2	41.0	87.0	83.5	82.0	87.9	27.1	32.5
1400	138.1	112.4	24.5	51.2	87.0	74.5	84.6	85.5	28.2	34.2
1600	144.9	118.8	35.1	57.3	84.8	65.5	87.1	83.0	30.8	34.3
1800	148.9	124.0	40.4	63.9	78.3	55.7	84.5	80.9	30.9	34.5
2000	162.4	130.5	47.9	71.7	56.5	45.9	76.4	79.6	27.0	35.6
2200	190.3	132.9	59.6	74.5	39.1	42.0	74.2	79.1	24.8	36.0

**Figure 2:**
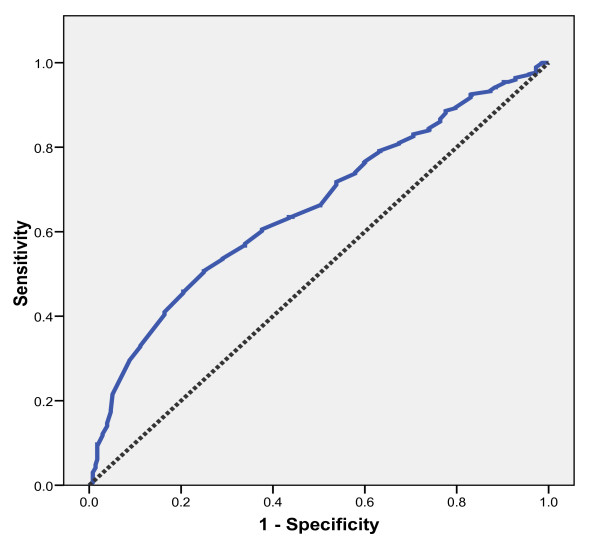
**ROC curve with sensitivity and 1-specificity of TLC cut-off values identifying a CD4 count of < 200 cells/mm^3 ^(AUC = .66)**.

Considering the best cut-off values of TLC, that are with the highest sensitivity and specificity combinations, a TLC ≤ 1780 cells/mm^3 ^was found to have maximal sensitivity (61%) and specificity (62%) for predicting a CD4 cell count of < 200 cells/mm^3^. Meanwhile, a TLC ≤ 1885 cells/mm^3 ^would identify only 59% of patients with CD4 count of < 350 cells/mm^3 ^(sensitivity, 59%; and specificity, 61%). The combined sensitivity and specificity for patients above 40 years of age was greater since their ROC curve AUC 0.72 was greater as compared to 0.64 of patients ≤ 40 years; the AUC was also slightly greater for female sex (0.66 versus 0.65). For subjects aged less than 18 years the best TLC cut-off was 2050 with sensitivity and specificity of 53.2% and 52.2%, respectively. The ROC curve (Figure 2) showed a fairly poor separation between classes (the diagonal reference line represents random performance).

## Discussion

According to the WHO's general principle to guide decision making about when to initiate ART in resource poor setting, a wider availability of CD4 testing is indispensable. However, the scarcity of this technology shouldn't be a cause to deter treatment while the patient's condition deteriorates if there is access to TLC and knowledge of clinical staging [4]. Several studies revealed reasonably adequate sensitivity and specificity to consider TLC as a surrogate measure for CD4 [5-10].

Nevertheless, this study supports the notion by Gupta and colleagues (2007), as we observed low sensitivity and specificity of TLC as an alternate marker to initiate ART. In our study, the sensitivity and specificity of TLC < 1200 to predict CD4 count < 200 for adults were 41% and 83.5%, and these figures were lower than that reported recently from India, 59% and 94%, respectively [11]. As it was reported by Jacobson and colleagues (2003), TLC may still be used in resource limited area with the understanding of its low sensitivity and specificity. Stebbing and colleagues also indicated that despite minimally less reliability of TLC as a surrogate for CD4, TLC is important tool in the absence of expensive equipment to measure CD4 [13].

We recommend further exploration of available data to ameliorate such disparities of sensitivities and specificities of TLC as proxy for CD4 count or else keep on expansion of access to CD4 counter.

We also recommend inclusion of white blood cells, red blood cells, haemoglobin, hematocrit and platelets in such analyses and also separate analysis for pregnant women, which we considered as the limitations of this manuscript.

## Competing interests

The authors declare that they have no competing interests.

## Authors' contributions

DD wrote the proposal, secured the funding and organized the data collection. EL analysed and interpreted the data and developed the manuscript. Both authors read and approved the final manuscript.
